# Reports on Hospitals of the United Kingdom

**Published:** 1914-03-07

**Authors:** Henry Burdett


### REPORTS ON

### Hospitals of the United Kingdom.

By SIR HENRY BURDETT, K.C.B., K.C.V.O.

SERIES III.

### THE NORTHAMPTON GENERAL HOSPITAL.

### The Board of Management and the Report in THE HOSPITAL of February 14, 1914 (page 535).

The Chairman of the Board of Management of the Northampton General Hospital has sent for publication a letter (see p. 618) on Sir Henry Burdett's Report, published in The Hospital of February 14. We append Sir Henry Burdett's reply:—

"Mr. Manfield's letter would not have been written had he and his colleagues appreciated the point of view from which these reports are prepared and written. It has been our responsibility to endeavour to ascertain how far each hospital inspected attains the standard of a modern, up-to-date hospital, and where, if anywhere, it fails at present to reach this test of the highest efficiency. The whole purport and aim of the reports is to help the managers of the hospitals, and the people who reside in the areas which each institution serves, to realise where they stand to-day, and in what way co-operation between the managers and the people for whose benefit the hospital exists, can add to the efficiency and usefulness of each hospital inspected. There is no thought or possibility of any personal question arising, and every recommendation made is based upon general principles, and rests upon the actual experience of the working of hospitals all over the country.

Mr. Manfield has evidently misapprehended or not appreciated these facts, or he would not speak of 'charges' in relation to the raising of funds, which, after all, means the best way to awaken an interest and to secure the free gifts of an ever-increasing number of people to the hospital every year. Again, he would not have come to such a conclusion, nor would any misunderstanding have arisen in the case of Mr. Loder, had they realised that the allusion to the not infrequent practice of periodically changing the Chairman of the Finance Committee of a hospital, say once in five years, was merely the affirmation of a practice which has proved fruitful in results and grateful to some of the most zealous chairmen and workers for the voluntary hospitals which the country possesses. No one can appreciate more fully and gratefully than we do the hard work and great personal services rendered to this hospital by several members of the committee, and especially by Mr. Loder, who we cannot forget took an active part from the outset in the proceedings which have secured the present hospital buildings. The increased revenue testifies to the good work accomplished, which we appreciate at its true value; but our ambition was and is to secure the maximum of efficiency for this county hospital by providing it without delay with an adequate income—that is, with an additional £2,000 a year—within the next six months. All the report insists upon is that until this £2,000 extra income is obtained 'those especially responsible for the financial work of the hospital have not fulfilled their responsibility adequately.' We intended nothing personal, and we regret if any words the report contained have conveyed the contrary impression to anyone. Our wish was to encourage and stimulate, not to censure. We hope, therefore, that Mr. Manfield and Mr. Loder will re-read the report in the spirit in which this explanation is given, and that any feeling of annoyance or irritation which may have arisen may so cease to exist.

There is surely no reason to speak of our vigorous insistence upon the needs of this hospital, and the way to meet them successfully, as 'a most serious charge preferred jointly against the Committee and the people of Northampton.' If the report is read carefully it will be seen that this is not so, for we were careful to point out that the criticism we heard during our visit, that the infirmary was always asking for money, did not reflect on the managers of the hospital or its medical staff; but really condemned those inhabitants of Northampton who were taking so little part in the support of the hospital to which they owe a deep debt of gratitude. And we went on to say that if every individual of Northampton could have been with us during our inspection, have seen the hospital as we saw it, with its excellent work, and its paramount value to the patients directly and to every resident in the town and county of Northampton indirectly, the next day would have seen hundreds of Northamptonians becoming subscribers to the County Hospital. It is not, therefore, a question of making charges against anyone, or of censuring anyone. The report merely represents the earnest endeavour of one who sees and knows the facts to make those facts known and realised by every Northamptonian whom they immediately concern.

This meaning, we are glad to see, was fully grasped by the editors of the Northampton papers. Thus, the editor of the *Northampton Mercury* of the 21st ultimo, in dealing with the need for an additional income of £2,000 for the County Hospital, writes: 'The town and county could without the least strain raise not only £2,000 but many times that if the burden were equally distributed. The trouble is that so many men of leisure will not give of their time, and so many men of wealth will not give of their money. Look upon the lists of those who are chiefly responsible for managing and financing voluntary institutions in Northampton, and you will see that the names of the hard workers and generous givers are very, very few in proportion to the number of well-to-do people. The chief service that could be rendered to Northampton town and county would be to touch the hearts of the many who can help but who do little or nothing.' In thanking the *Northampton Mercury* for so eloquently voicing our meaning and aim, we take courage and affirm once more that the extra £2,000 a year can be and, we believe, will be forthcoming providing the services of the younger men and of the women of Northampton can be won for the hospital, and set to work under the presidency of the Mayor and Mayoress, or some other influential men and women, without a moment's avoidable delay.

We are indeed sorry that any words we published should have been so seriously misapprehended. Our one desire is to secure that all may join hands in the good work of raising the income of the County Hospital to the required amount by a combined effort, which is sure to attain success, because it deserves to achieve it."

### Letter from the Chairman of the Northampton General Hospital.

The following is the letter of the Chairman of the Board of Management, which contains several points which Sir Henry Burdett thinks it will be most useful for him to deal with paragraph by paragraph:—

*To the Editor of* The Hospital.

Northampton General Hospital.
February 27, 1914.

Sir,—The Board of Management of the Northampton General Hospital feel that some answer is required to the article published by Sir Henry Burdett in The Hospital on the 14th inst. Whilst the Board welcome criticism of the work of the institution under their care, they cannot admit the justice of many of the charges which have been made. Undoubtedly the most serious charge is that preferred jointly against "The Committee and the people of Northampton," who, according to Sir Henry Burdett, "have so far failed to realise fully the claims and needs of the General Hospital." The Finance Committee and their Chairman are specially censured, since Sir Henry observes that "those responsible for the financial work of the hospital have not fulfilled their responsibility adequately."

### The Increased Revenue.

In view of such a sweeping statement what are the facts? That during the past eight years, since the rebuilding of the hospital, the annual receipts have risen from £8,666 to £12,132, an increase of £3,466, practically 40 per cent. This result has been largely brought about by appeals to the public through the Board of Management, upon the advice of the Finance Committee. Whilst it is in no sense the duty of the Chairman of the Finance Committee to collect subscriptions, Mr. Loder has himself on several occasions sent out strong personal appeals and has made special efforts to secure new subscribers, with most satisfactory results. The Hospital Week Committee have also rendered invaluable help by working up their annual contributions from £1,229 in 1905 to £2,225 in 1913, an increase of £926.

[We were quite aware and rejoiced to see that the total receipts from revenue under all heads showed a considerable increase in the eight years. Unfortunately the accounts as they are at present rendered, although it was stated on page 12 of the report for 1905 that the Uniform System of Accounts had been adopted, do not in fact conform to the Uniform System. They omit to include the legacies; they omit to make the totals on the income side of the accounts agree with the actual sums acknowledged as received from contributors in the shape of annual subscriptions, donations, and so forth, as acknowledged in the report, or to give the total of the actual sums acknowledged, and make that total agree with the figures in the abstract of accounts. There is no balance sheet, which is one of the most important features in the Uniform System, but merely a balance of revenue account. Further, the totals given in the abstract of accounts are not always easy to trace in the report. The item of donations, for example, which is given in the accounts published in the report for 1913 as £823 11s. 2d., does not agree with the sum acknowledged under Benefactors on page 136 of the report. Whether it agrees with the total of the sums acknowledged as donations and individual amounts on pages 66 to 103 does not appear, as no totals, audited or otherwise, are given. We observe, too, that under the head of subscriptions appears "The Hospital Week Committee for 'letters.'" Does that mean money paid for actual "letters" received and used by patients? If so, the Uniform System would not credit this money to annual subscriptions. The actual contribution from the Hospital Week Committee in 1913 was £2,225, which in justice ought to be clearly shown in one total in the accounts. It was for these reasons that we did not go into the question of the increase in revenue, because the sources from which that revenue was derived, apart from the Hospital Week collections, cannot be accurately ascertained from the report and accounts as at present rendered. In congratulating the management upon the fact that there has been this increase in revenue, we must urge that the important point to remember is that the hospital urgently needs £2,000 a year more income to enable it to do its full work with the maximum of efficiency.]

### A Misunderstanding.

Unfortunately since the publication of Sir Henry Burdett's Report, Mr. Loder, who for many years has so generously given his time and money, considers the criticism so unjust that he has felt it incumbent upon himself to resign his position on the Committee. The Board of Management, realising the serious loss this would be to the hospital, have earnestly requested him to withdraw his resignation. He, Mr. Loder, and the Finance Committee enjoy the entire confidence of the Governors of the institution, and the Board regard the suggestion put forward, that the Chairman of that Committee should be periodically changed, as an innovation which, if adopted, would adversely affect the interests of the hospital.

[We exceedingly regret that our suggestion, based upon experience, should have given offence to anybody. We wish it to be clearly understood that we were fully alive to the fact that circumstances alter cases, and that it is clearly a question for Mr. Loder, with the Committee and the Governors, to determine whether or not the advice tendered should be acted upon, so far as this hospital is concerned. In such circumstances it will be seen there is nothing personal in the suggestion.]

On some of the minor points of Sir Henry Burdett's Report the Board make the following observations:—

### Pay of Sisters and Nurses.

On this question Sir Henry states:—

"It is hardly credible, but it is a fact, that the remuneration offered to the sisters in the wards commences at £28 per annum, a sum at which it has stood for very many years. No other hospital of its size and reputation that we have discovered offers its sisters less than £30, and most of them give £35, rising to £40 per annum."

He speaks of the sisters' remuneration commencing at £28, but does not mention that their salaries rise by £2 a year to £36. In his report on the Bedford Hospital, which he visited just before coming to Northampton, Sir Henry omits to state that the sisters' salaries commence at £28 and rise to £35. In the neighbouring hospital of Leamington the sisters' salaries commence at £25, rising to £35.

[We stated, with a full knowledge of the average rate of salaries in hospitals, that no other hospital of its size and reputation that we have discovered offers its sisters less than £30, and most of them give £35, rising to £40 per annum. Mr. Manfield quotes the case of Bedford and Leamington, but they are hospitals of a different type from the Northampton General Hospital. The average number of beds daily occupied at the Northampton General Hospital is 154; at Bedford, 76; and at Leamington, 74.65. Mr. Manfield must see that he has not done justice to his own institution by these comparisons, or to the standard it should maintain, if we may say so without offence.]

### Some Shocking Lavatories.

This relates to the wash-basins, and there is no doubt that those in the wardmaids' dormitory are of a very old pattern, and that three are broken or missing. This had been realised, and it had already been proposed to clear them all away and put in modern ones. Sir Henry appears to suggest that these lavatories are objectionable from a "moral point of view." The moral point of view, it must be presumed, means that the basins are all in one row and that each basin is not screened off; therefore the maids have to wash in each other's presence. The lavatory, however, is shut off from the sleeping cubicles, and there is also a separate bathroom and w.c. provided. Sir Henry forgets that the basins (six in number) in the new servants' dormitory, which are included in his condemnation, were put there by the Building Committee of which he was a member. These basins, from a moral point of view, are exactly similar to those in the wardmaids' dormitory. All these premises are visited by the matron once a week or oftener.

[We must maintain our criticisms here, and if we have any blame for having allowed our name to appear upon the Building Committee, if that is the point, we can only express our sorrow and regret for any share we may have indirectly had in a state of affairs which ought never to have been, and which certainly should be remedied without a moment's avoidable delay. The shut-off referred to does not lessen the moral objection to the arrangement as it stands at present.]

### Handling and Distribution of Coal.

Sir Henry did not see this in operation, and appears to have been entirely misled by some informant. He writes a strong paragraph on what he calls "the old and archaic system" in vogue, and of which the medical staff "bitterly complain." The system originally adopted was undoubtedly noisy and objectionable. The noise was caused by wheeling the trolleys up the incline from the basement, and by the rattling of the tins upon the trolleys, but this system was discontinued some two years ago by the opening of a new coal store on the level and by improving the trolleys, which, contrary to Sir Henry's statement, have always had rubber-tyred wheels.

[Mr. Manfield does not deal with our main contentions or remove the chief objections which we pointed out. If the Committee have any doubt still, we would suggest they should become in-patients of the hospital when they are next seriously ill, and experience in their own person what the patients in the general wards may and do suffer, from the present noisy system of distributing the coal. Rubber-tyred wheels are evidently not a preventive of all noise in the present case, and it would appear, as we stated, that the trolleys are not suitable, nor provided with wheels or tyres which enable the silent distribution of the coal which prevails in the best-administered hospitals.]

### What the Hospital Needs.

Under this heading Sir Henry writes:—

"The facts that there is such an overflowing attendance at the out-patients' department, and that there is a growing increase in the number of in-patients, seem to warrant an inquiry by the Committee into the number of insured persons treated as out-patients, to ascertain whether their treatment does not properly devolve upon their panel doctors rather than upon the hospital authorities. In any case, there is urgent need for the appointment of a sister to look after the out-patient work, and there is also need for a small theatre in connection with the out-patient department."

Nearly all hospitals have found that the effect of the Insurance Act has been to increase the demand for in-patient treatment. The members of the medical staff have power to refuse treatment to insured out-patients who can derive equal benefit from their panel doctors. The number of out-patients has decreased by 309 during the past year; in the previous year there had been an increase of 1,200. There has been a sister in charge of the out-patient department for the past four years. The operation theatre sister assists the out-patient sister with minor operations in that department one morning in each week.

[We hope a small committee will be appointed, as we suggested. Mr. Manfield admits that at the present time the sister of the operation theatre has to share the out-patient work, and inferentially that the out-patients needing operations are put into the operation theatre. We can only repeat that such a practice is wrong hygienically and administratively, and we hope it will be put an end to.]

The New Electrical and $x$-Ray Department.

It is but fair to Sir Henry to say that he considers "the general aspect of the hospital distinctly satisfactory," and that "the people of the town and county of Northampton should be proud of it as it exists to-day." He bestows praise on the new laundry, with certain reservations, upon the new mortuary, the kitchen and stores department, and also on the electrical and $x$-ray department, but in regard to the latter it may be news to Sir Henry that since his visit it has been decided, upon the recommendation of the Medical Board, guided by an expert, to spend £184 to bring that department up to date.

[This is not a surprise, but merely an endorsement of our statement that Dr. Robson has a thorough and unusual knowledge of this important section of hospital work.]

The Board are content to leave it to "the people of Northampton" as to whether Sir Henry Burdett's criticism of the hospital is fully justified or not. They cannot, however, associate themselves with the suggestion that the hospital has received such inadequate support as Sir Henry appears to suggest. At the same time the fact should be generally realised that the ever-advancing demands of medical science, and the consequent expense entailed, require that increasing financial aid should be forthcoming. If Sir Henry Burdett's Report will further stimulate the generosity of the public it will have served a valuable purpose.

Yours faithfully,
H. Manfield,
Chairman of the Board of Management.

[We sincerely hope with Mr. Manfield that our report may further stimulate the generosity of the public, and that the £2,000 of additional income may speedily be supplied.]

**Owing to pressure upon our space the Report on "Some Cottage Hospitals" has been unavoidably held over.**

